# Efficacy of Abiraterone and Enzalutamide in Pre- and Postdocetaxel Castration-Resistant Prostate Cancer: A Trial-Level Meta-Analysis

**DOI:** 10.1155/2017/8560827

**Published:** 2017-11-21

**Authors:** Mike Fang, Mary Nakazawa, Emmanuel S. Antonarakis, Chun Li

**Affiliations:** ^1^Department of Population and Quantitative Health Sciences, Case Western Reserve University School of Medicine, Cleveland, OH, USA; ^2^University of Kentucky College of Medicine, Lexington, KY, USA; ^3^Departments of Oncology and Urology, Johns Hopkins University School of Medicine, Baltimore, MD, USA

## Abstract

We examined the comparative efficacies of first-line abiraterone and enzalutamide in pre- and postdocetaxel settings in castration-resistant prostate cancer (CRPC) through a trial level meta-analysis. A mixed method approach was applied to 19 unique studies containing 17 median overall survival (OS) estimates and 13 median radiographic progression-free survival (PFS) estimates. We employed a random-effects meta-analysis to compare efficacies of abiraterone and enzalutamide with respect to OS and PFS. In the predocetaxel setting, enzalutamide use was associated with an increase in median OS of 5.9 months (*p* < 0.001), hazard ratio (HR) = 0.81, and an increase in median PFS of 8.3 months (*p* < 0.001), HR = 0.47 compared to abiraterone. The advantage of enzalutamide improved after adjusting for baseline Gleason score to 19.5 months (*p* < 0.001) and 14.6 months (*p* < 0.001) in median OS and PFS, respectively. In the postdocetaxel setting, the advantage of enzalutamide use was nominally significant for median PFS (1.2 months *p* = 0.02 without adjustment and 2.2 months and *p* = 0.0007 after adjustment); there was no significant difference in median OS between the two agents. The results from this comprehensive meta-analysis suggest a survival advantage with the use of first-line enzalutamide over abiraterone in CRPC and highlight the need for prospective clinical trials.

## 1. Introduction

Prostate cancer is the most common cancer diagnosis in men and is projected to account for more than 160,000 new diagnoses in the United States in this year [1]. While localized prostate cancer has excellent prognosis, castration-resistant prostate cancer (CRPC) is uniformly lethal after a period of about 1–3 years [2]. Castration resistance represents the cumulative result of escape mechanisms deployed by the tumor to overcome androgen deprivation [3], clinically manifested by biochemical or radiographic progression despite castrate levels of serum testosterone [4]. In recent years, the FDA approvals of several new therapeutic agents for CRPC, such as the androgen receptor signaling axis-targeting agents, abiraterone [5] and enzalutamide [6], have transformed the clinical management of advanced prostate cancer. Despite these advancements, the improvements in survival offered by these therapies are modest, on the order of several months, given the rapid and inevitable emergence of resistance. The issue of resistance is particularly relevant in CRPC with the rise of cross-resistance between abiraterone and enzalutamide [7–10], as well as between these agents and taxane therapies [11].

The availability of multiple CRPC therapies necessitates an understanding of optimizing the sequence in which these therapies are deployed, an area of major investigative effort [12, 13]. Abiraterone and enzalutamide were initially approved for use in the postdocetaxel setting [14, 15], with expanded indications for docetaxel-naïve CRPC [16–18] use shortly thereafter. Given their favorable side effect profile and convenience of outpatient therapy compared to taxanes, abiraterone and enzalutamide are often deployed early in the CRPC disease course, with much of the decision-making as to which agent should be initiated, deferred to oncologist experience, toxicity profile, and patient preference. To date, there have been no direct prospective investigations comparing the efficacies of abiraterone and enzalutamide in CRPC patients, with only a few retrospective analyses [19] of single institution experiences.

Given that the emergence of cross-resistance and unique selective pressures exerted by abiraterone and enzalutamide may influence the efficacies of successive treatments, there is a need to determine whether these two therapies are truly interchangeable entities or whether there are differences in survival outcomes. We thus conducted a comprehensive trial-level meta-analysis to examine the comparative efficacies of abiraterone and enzalutamide in the first-line CRPC (predocetaxel) and second-line CRPC (postdocetaxel) settings by utilizing the published literature.

## 2. Methods

The primary goal of this study is to compare median overall survival (OS) and median radiographic progression-free survival (PFS) of abiraterone and enzalutamide in both pre- and postdocetaxel settings. The treatment sequences examined were first-line abiraterone (denoted as AX) and first-line enzalutamide (EX) in the predocetaxel setting and docetaxel-to-abiraterone (DA) and docetaxel-to-enzalutamide (DE) in the postdocetaxel setting. The median time-to-event estimates are defined as the time from the start of the treatment of interest, abiraterone, or enzalutamide, to the time at which 50% of the subjects in the study group have reached the outcome (death for OS, and disease progression for PFS). Disease progression is determined by radiographic evidence based on the RECIST [20] and PCWG2 [21] criteria.

**Figure 1:**
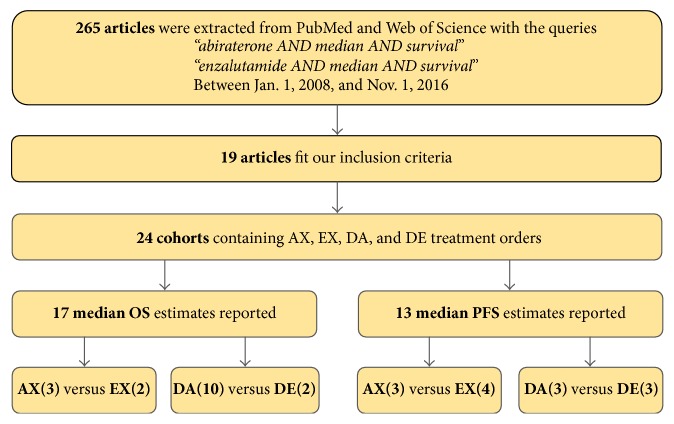
Flow schematic depicting inclusion and exclusion criteria for selection of studies. AX: predocetaxel abiraterone; EX: predocetaxel enzalutamide; DA: postdocetaxel abiraterone; DE: postdocetaxel enzalutamide; OS: overall survival; PFS: radiographic progression-free survival.

Using the Entrez [22] package for Python, PubMed and Web of Science were queried initially for “abiraterone AND median AND survival” and “enzalutamide AND median AND survival” with a date range from January 1, 2008, to November 1, 2016 (Figure 1). Querying through “median” estimates allowed us to maximize the number of suitable studies. Many studies had only a single cohort that met our inclusion criteria (described below), which may have been omitted under other queries such as “hazard ratio.” A total of 265 peer-reviewed articles, in English, were curated. Studies were excluded if any of the following conditions were met: (1) greater than 20% of the study population utilized a prior therapy with another androgen deprivation agent (e.g., a DA study in which greater than 20% of patients had prior enzalutamide use); (2) no confidence intervals or bounds were reported for the median estimates; (3) disease progression in a PFS study was not evaluated through radiographic evidence; studies with composite radiographic and PSA progression estimates were also excluded. For studies with a series of publications, we use the most up-to-date estimates in our analyses. To assess the potential of publication bias, we generated funnel plots of effect against precision for all treatment sequences.

In total, 6 clinical trials and 18 nonclinical trials (Table 1) qualified for inclusion using our criteria: 17 cohorts provided median OS estimates while 13 cohorts provided median PFS estimates. Of the 17 median OS estimates, 3 were for the AX sequence and 2 were for EX, and 10 were for DA and 2 were for DE. Of 13 cohorts with median PFS estimates, 3 were for the AX sequence and 4 were for EX and 3 were for DA and 3 were for DE (Figure 1). Available confidence intervals or bounds and sample sizes were also recorded. Most, but not all, of these studies also reported summary baseline characteristics such as race, mean or median age, proportion of patients with a Gleason score ≥ 8, and median baseline PSA score (ng/mL) (Table 1). In addition to the median time-to-event estimates, most studies we considered reported the proportion of patients with PSA decline ≥ 50% (Table 1). Unequal variance *t*-tests were performed for these characteristics (Table 2).

Due to a lack of access to individual-level data, a trial-level meta-analysis was conducted using the rma.mv() function in the metafor package of R software [23]. Heterogeneity within treatment sequence groups (*I*^2^ > 80% except for the DA sequence with respect to PFS) suggested a mixed-effects model when combining effect sizes. The median time-to-event estimate from each study was weighted by its inverse variance, where the variance was calculated from the reported 95% confidence interval (CI) as (UB − LB)/3.92, where UB and LB are the upper and lower bounds of the CI, respectively. Six studies only reported a lower bound presumably because early censoring prevented estimation of the upper bound; in these cases, the variance was calculated as (median-LB)/1.96. The rma.mv() function in the metafor package accounts for nonindependence in observed effects as we analyzed more than one median estimate from several studies (Ferraleschi et al., Higano et al., Poon et al., Thortzen et al., and Yamasaki et al.). This function also allows adjustment for baseline patient characteristics such as the proportion of patients with a Gleason score ≥ 8.

For each of the two time-to-event outcomes (OS and PFS), a hazard ratio (HR) between two treatment sequences was calculated as the inverse ratio between their median survival estimates. It has been shown [24] that there is a high concordance between HR and inverse median survival ratio. In fact, under the exponential survival model (i.e., constant hazard function model), HR equals the inverse ratio of median survival time.

## 3. Results

As described in Methods and Figure 1, 265 publications were manually assessed and 24 cohorts met our inclusion criteria. Although we cannot rule out publication bias or lack thereof due to the limited number of studies per group, we do not discern any bias in the funnel plots (Supplemental S1 in Supplementary Material available online at https://doi.org/10.1155/2017/8560827).

The baseline characteristics—age, proportion with Gleason score ≥ 8, and baseline median PSA score—were comparable between AX and AE and between DA and DE for both OS and PFS analyses (Table 2). As expected, there was a negative correlation between the proportion of patients with a Gleason score ≥ 8 and median baseline PSA score for both OS (*r* = −0.22, *p* = 0.45) and PFS (*r* = −0.44, *p* = 0.28) (Supplemental Figures S2 and S3). The DE cohorts had a significantly higher proportion of patients with PSA decline ≥ 50% than the DA cohorts for both OS and PFS (Table 2). The EX cohorts also had a higher proportion of patients with PSA decline ≥ 50% than the AX cohorts, and the difference was significant for PFS (Table 2). The PSA advantages of enzalutamide over abiraterone are consistent with our main results with respect to median survival.

**Figure 2:**
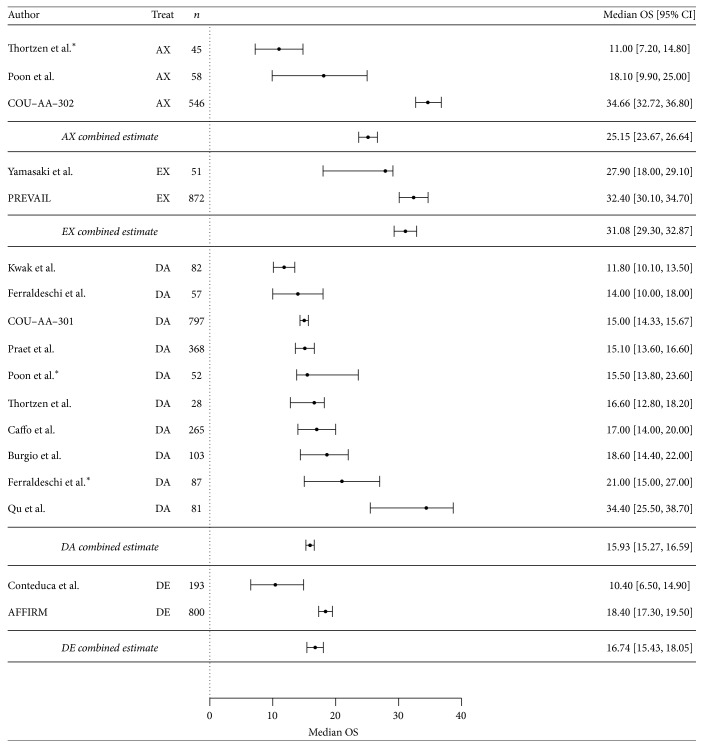
Forest plot depicting median OS (in months) of AX (*n* = 3), EX (*n* = 2), DA (*n* = 10), and DE (*n* = 2) cohorts. Open circles on confidence bounds denote studies that only provided the lower confidence bound.

The combined estimates for median OS for all four treatment sequences are shown in Figure 2. The median OS for EX (31.1 months, 95% CI 29.3–32.9) was significantly longer than that for AX (25.2 months, 95% CI 23.7–26.6). The difference was 5.9 months (*p* < 0.0001; HR = 0.81). Because the studies had different baseline characteristics especially with respect to the proportion of patients with a Gleason score ≥ 8, we also performed an analysis to adjust for this baseline characteristic. The adjusted result showed an even larger difference in outcomes between the AX and EX sequences, with the EX group having a 19.5 month improvement in OS (95% CI: 16.50–22.53) compared to the AX group (*p* < 0.001) (Table 3). However, there was little difference in median OS between DA (15.9 months, 95% CI 15.3–16.6) and DE (16.7 months, 95% CI 15.4–18.1) (*p* = 0.28); the HR was 0.95.

**Figure 3:**
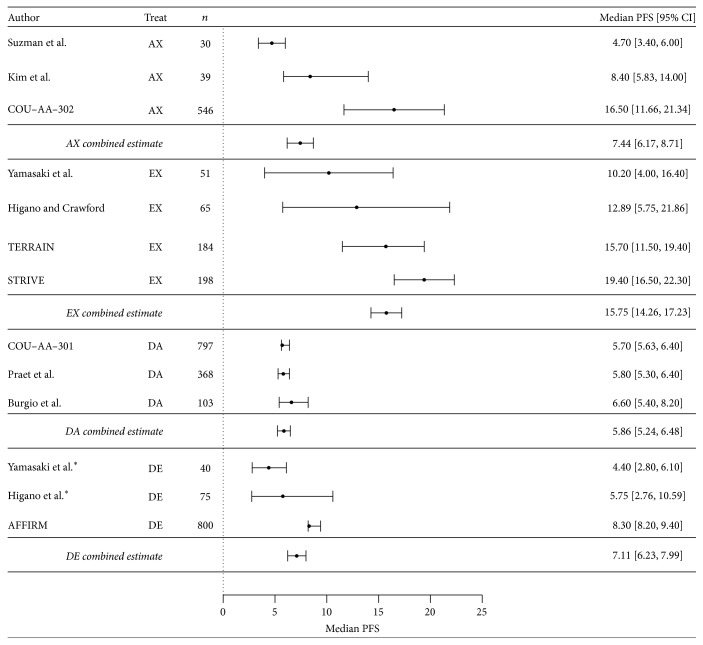
Forest plot depicting median radiographic PFS (in months) for AX (*n* = 3), EX (*n* = 4), DA (*n* = 3), and DE (*n* = 3) cohorts.

The combined estimates for median PFS for all four treatment sequences are shown in Figure 3. The patterns are similar to those for OS. The median PFS for EX was 15.8 months (95% CI 14.3–17.2), while that for AX was 7.4 months (95% CI 6.2–8.7), showing a significance difference of 8.3 months between the sequences (*p* < 0.0001). The corresponding HR was 0.47. The advantage of EX is also increased after adjusting for the baseline Gleason score, representing a 14.6-month improvement compared to the AX group (*p* < 0.001) (Table 3). There was a nominally statistical difference in median PFS between DA (5.9 months, 95% CI 5.2–6.5) and DE (7.1 months, 95% CI 6.2–8.0) (1.2 months; *p* = 0.02); the HR was 0.82. The adjustment for the baseline Gleason score also made the difference between DE and DA more significant, with DE having a 2.2-month improvement over DA in PFS (*p* = 0.0007).

## 4. Discussion

In this meta-analysis, we compared the efficacies of abiraterone and enzalutamide by pooling results from 19 published studies, which yielded 24 cohorts with median OS and/or median PFS estimates. We found that treatment with first-line enzalutamide was associated with improved outcomes both in terms of OS (HR = 0.81) and PFS (HR = 0.47) compared to first-line abiraterone in the predocetaxel CRPC setting. First-line enzalutamide treatment was associated with a median OS advantage of 5.9 months and a median PFS advantage of 8.3 months; these advantages were further improved to 19.5 and 14.6 months, respectively, after baseline Gleason score was taken into account. We note that the greatest median survival estimates for AX belonged to the clinical trial COU-AA-302 for both OS and PFS and that, in the case of OS, COU-AA-302 showed much longer median survival than all other studies which, we suspect, is due to the highly selective nature of clinical trial investigations. The single-center studies that decrease the combined estimate for predocetaxel abiraterone may be more reflective of real-world experiences. In the postdocetaxel setting, enzalutamide showed a small but statistically significant (especially after adjusting for baseline Gleason score) advantage over abiraterone with respect to PFS.

A recent pooled analysis of only major phase III clinical trials PREVAIL, AFFIRM, COU-AA-301, and COU-AA-302 conducted by Chopra et al. [25] yielded similar but less significant findings. Enzalutamide was suggested to be superior to abiraterone with respect to radiographic PFS in both pre- and postdocetaxel settings; their results for the OS were not statistically significant although the direction of effect was in agreement with our findings. In that study, adjustments for baseline measures were not considered even though Gleason score has been shown to be strongly predictive of survival outcomes in CRPC [26]. Our finding of the association between enzalutamide use and longer survival for both OS and radiographic PFS, especially after adjusting for baseline Gleason score, underscores the need for prospective studies comparing the two drugs and suggests that abiraterone and enzalutamide should perhaps not be considered as interchangeable AR-targeting agents.

It is important to note that our study seeks to identify differences in outcomes of abiraterone and enzalutamide utilized in the CRPC trajectory but does not directly address the issue of optimal sequencing of AR therapies in relation to one another or with docetaxel. We were also unable to directly compare efficacies of one AR agent after the other (i.e., abiraterone after enzalutamide or vice versa) given the lack of studies on these sequences fitting our inclusion criteria. To date, there have been some retrospective studies reporting single-center experiences with the sequencing of abiraterone and enzalutamide. Maughan et al. [19] suggested enhanced PFS using the abiraterone-to-enzalutamide sequence over the enzalutamide-to-abiraterone sequence, suggesting that the former may maximize the therapeutic benefit of both therapies while minimizing cross-resistance. A second retrospective study also revealed similar findings [27]. Our finding of potential enzalutamide superiority in the first-line CRPC setting is not necessarily at odds with those results, as the AX and EX cohorts received a heterogeneous set of therapies after abiraterone or enzalutamide failure, and does not necessarily reflect outcomes for when one AR agent is followed directly by the other. In addition, due to the lack of access to individual patient-level data from the studies, we were unable to identify the subgroups of patients that benefited most from enzalutamide in the first-line setting. It is entirely possible that certain unknown patient characteristics are accounting for the superior survival in predocetaxel enzalutamide-treated patients. Mechanistically, these patients may have derived more benefit from first-line enzalutamide given that, perhaps, CYP17-driven adrenal androgen production (target of abiraterone therapy) was not the major driver of their disease.

Our findings, combined with the fact that several studies have suggested an attenuated response to the second AR agent compared to treatment naïve cases [7–10], are reasons to pursue prospective trials aiming to optimize treatment sequence in CRPC. To this end, the optimal sequencing of AR-targeting agents in CRPC is being assessed by ongoing prospective studies such as NCT02125357, a phase II randomized study of abiraterone-to-enzalutamide versus enzalutamide-to-abiraterone in chemo-naïve CRPC patients. Preliminary results suggest that enzalutamide use is associated with superior PSA response compared to abiraterone use first line [28]. This ongoing study also includes efforts on biomarker identification using circulating tumor DNA (ctDNA) to assess genomic alterations in genes such as AR, p53, and BRCA. Such predictive biomarkers are an important asset to clinical decision-making and treatment selection in the era of noninvasive tumor profiling. To that end, AR splice variant-7 (AR-V7) is a ligand-independent variant of the androgen receptor that has emerged as both an underlying mechanism of resistance and a promising predictive biomarker in CRPC. While there are over 20 known AR splice variants, AR-V7 has established clinical relevance with its detection in clinical specimens associated with inferior responses to abiraterone and enzalutamide [29–31]. Taken together, the comparative efficacy of abiraterone and enzalutamide must be assessed in relation to known and emerging biomarkers of resistance in CRPC.

Clinical decisions on the sequencing of therapies in CRPC remain largely consensus-based rather than evidence-based, given the lack of prospective head-to-head trials assessing efficacies of agents in relation to the sequence in which they are deployed. Here, we present a trial-level meta-analysis using data from prospective trials and retrospective studies, suggesting that enzalutamide use is associated with longer median OS and PFS compared to abiraterone in the first-line (predocetaxel) setting and that this survival improvement is further accentuated when baseline Gleason score is taken into account. These findings highlight the limitations in using a consensus-based approach to treatment selection in treatment naïve CRPC patients and the need to pursue prospective trial validation. However, until further work is done to confirm optimal treatment selection and treatment sequencing, biomarkers in the management of metastatic CRPC, clinical factors such as comorbid conditions, cost considerations, patient preference, and side effect profiles should continue to guide the clinician's decision on treatment sequencing of systemic therapies for men with metastatic CRPC.

## Supplementary Material

Supplemental 1, Figure: Funnel plots showing the effect against precision for all treatment sequences for OS/PFS.
Supplemental 2/3, Figure: Pearson correlations between median OS/PFS and other variables shown on the lower left with corresponding pairwise plots on the upper right. Distributions of each variable are displayed along the diagonal. Plotting PSA decline, Gleason score, and baseline PSA against median OS/PFS indicate trends that are consistent with expected clinical outcomes.
Supplemental 4/5, Appendix: Results of regression models for median OS/PFS as outcomes. Hash marks indicate the specific model.

## Figures and Tables

**Table 1:** All studies included in analysis.

Author/study	PMID	Clinical trial code	*n*	Treatment	Race	Age	Gleason score ≥ 8	Baseline PSA (median)	PSA decline ≥ 50%
*Thortzen et al.*	26971191		45	AX	White	71.3	60%	156	51%
*Poon et al.*	27001043		58	AX	Asian	77.0	28%	212	62%
*Suzman et al.*	25053178		30	AX	White	70.6	48%	192	34%
*Kim et al.*	25336698		39	AX	White	71.0		48.5	41%
*COU-AA-302*	23228172	NCT00887198	546	AX	White	70.5			29%
*Yamasaki et al.*	26722066		51	EX	Asian	74.0	78%	11.2	63%
Higano and Crawford	25698064		65	EX	White	68.0		35	63%
*TERRAIN*	26774508	NCT01288911	184	EX	White	70.3	55%	21	82%
*STRIVE*	26811535	NCT01664923	198	EX	White	72.0	51%	11	81%
*PREVAIL*	25888263	NCT01212991	872	EX	White	71.3	51%	54.1	78%
*Kwak et al.*	25099185		82	DA	Asian	71.0	74%	124.3	49%
*Ferraldeschi et al.*	25454616		57	DA	White	66.0	41%	155	32%
*COU-AA-301*	23142059	NCT00638690	797	DA	White	69.0	51%	27	38%
*Praet et al.*	26850781		368	DA	White	73.0		103	37%
*Poon et al.* ^*∗*^	27001043		52	DA	Asian	66.0	56%	191	50%
*Thortzen et al.* ^*∗*^	26971191		28	DA	White	70.7	71%	169	18%
*Caffo et al.*	24988879		265	DA	White	73.0	51%	86	50%
*Burgio et al.*	24999168		103	DA	White	74.0	53%	32.5	
*Ferraldeschi et al.* ^*∗*^	25454616		87	DA	White	69.0	55%	237	43%
*Qu et al.*	27489290		81	DA	White		49%	16.4	
*Conteduca et al.*	27434372		193	DE	White	73.1			51%
*Yamasaki et al.* ^*∗*^	26722066		40	DE	Asian		83%	23	44%
Higano and Crawford^*∗*^	25698064		75	DE	White	68.0		64	53%
*AFFIRM*	22894553	NCT00974311	800	DE	White	68.8	50%	107.7	54%

The studies that provided two cohorts for our analyses are denoted (^*∗*^) and correspond to those in Figures 2 and 3. Race is the predominant proportion within each cohort and age is either a mean or median measure. Blank cells indicate a lack of reporting in that category.

**Table:** (a) Studies reporting median OS estimates

	AX	EX		DA	DE		Overall
Studies (count)	3	2		10	2		17
Sample Size	649	923		1920	993		4485
Race (count)							
White	2	1		8	2		13
Asian	1	1		2	0		4
Age (median)	71.3	72.7		70.7	71.0		71.2
Baseline PSA (median)	184.0	32.7	*p* = 0.06	113.6	107.7	*∗*	107.7
Gleason score ≥ 8 (proportion)	42%	52%	*p* = 0.44	53%	50%	*∗*	39%
PSA decline ≥ 50% (proportion)	34%	77%	*p* = 0.16	36%	53%	*p* = 0.02	48%

**Table:** (b) Studies reporting median PFS estimates

	AX	EX		DA	DE		Overall
Studies (count)	3	4		3	3		13
Sample size	615	498		1268	915		3296
Race (count)							
White	3	3		3	2		11
Asian	1	1		2	0		2
Age (median)	70.6	71.2		73.0	68.4		70.6
Baseline PSA (median)	120.3	16.1	*p* = 0.39	32.5	64.0	*p* = 0.77	33.8
Gleason score ≥ 8 (proportion)	48%	56%	*∗*	51%	52%	*p* = 0.54	52%
PSA decline ≥ 50% (proportion)	30%	77%	*p* = 0.003	38%	53%	*p* = 0.06	47%

*T*-tests with unequal variances *p* values are shown for the comparisons between AX and EX and between DA and DE. ^*∗*^Not enough information for the test.

**Table 3:** Enzalutamide advantage over abiraterone (without and with adjustment for baseline Gleason score) in months (*p* value).

	Unadjusted	Adjusted
OS		
Predocetaxel	5.9 (<0.001)	19.5 (<0.001)
Postdocetaxel	0.8 (0.28)	1.5 (0.7)
PFS		
Predocetaxel	8.3 (<0.001)	14.6 (<0.001)
Postdocetaxel	1.2 (0.02)	2.2 (<0.001)

More detailed results are available in Supplemental S4 and S5.

## References

[1] Siegel R. L., Miller K. D., Jemal A. (2017). Cancer statistics, 2017. *CA: A Cancer Journal for Clinicians*.

[2] Halabi S., Lin C.-Y., Kelly W. K. (2014). Updated prognostic model for predicting overall survival in first-line chemotherapy for patients with metastatic castration-resistant prostate cancer. *Journal of Clinical Oncology*.

[3] Nakazawa M., Paller C., Kyprianou N. (2017). Mechanisms of Therapeutic Resistance in Prostate Cancer. *Current Oncology Reports*.

[4] Higano C. S., Crawford E. D. (2011). New and emerging agents for the treatment of castration-resistant prostate cancer. *Urologic Oncology: Seminars and Original Investigations*.

[5] Attard G., Reid A. H. M., Yap T. A. (2008). Phase I clinical trial of a selective inhibitor of CYP17, abiraterone acetate, confirms that castration-resistant prostate cancer commonly remains hormone driven. *Journal of Clinical Oncology*.

[6] Ning Y.-M., Brave M., Maher V. E. (2015). U.S. food and drug administration approval summary: Enzalutamide for the treatment of patients with chemotherapy-naïve metastatic castration-resistant prostate cancer. *The Oncologist*.

[7] Bianchini D., Lorente D., Rodriguez-Vida A. (2014). Antitumour activity of enzalutamide (MDV3100) in patients with metastatic castration-resistant prostate cancer (CRPC) pre-treated with docetaxel and abiraterone. *European Journal of Cancer*.

[8] Loriot Y., Bianchini D., Ileana E. (2013). Antitumour activity of abiraterone acetate against metastatic castration-resistant prostate cancer progressing after docetaxel and enzalutamide (MDV3100). *Annals of Oncology*.

[9] Nadal R., Zhang Z., Rahman H. (2014). Clinical activity of enzalutamide in docetaxel-naïve and docetaxel-pretreated patients with metastatic castration-resistant prostate cancer. *The Prostate*.

[10] Badrising S., van der Noort V., van Oort I. M. (2014). Clinical activity and tolerability of enzalutamide (MDV3100) in patients with metastatic, castration-resistant prostate cancer who progress after docetaxel and abiraterone treatment. *Cancer*.

[11] Van Soest R. J., De Morrée E. S., Kweldam C. F. (2015). Targeting the androgen receptor confers in vivo cross-resistance between enzalutamide and docetaxel, but not cabazitaxel, in castration-resistant prostate cancer. *European Urology*.

[12] Valenca L. B., Sweeney C. J., Pomerantz M. M. (2015). Sequencing current therapies in the treatment of metastatic prostate cancer. *Cancer Treatment Reviews*.

[13] Sartor O., Gillessen S. (2014). Treatment sequencing in metastatic castrate-resistant prostate cancer. *Asian Journal of Andrology*.

[14] de Bono J. S., Logothetis C. J., Molina A. (2011). Abiraterone and increased survival in metastatic prostate cancer. *The New England Journal of Medicine*.

[15] Scher H. I., Fizazi K., Saad F. (2012). Increased survival with enzalutamide in prostate cancer after chemotherapy. *The New England Journal of Medicine*.

[16] Ryan C. J., Smith M. R., Fizazi K. (2015). Abiraterone acetate plus prednisone versus placebo plus prednisone in chemotherapy-naive men with metastatic castration-resistant prostate cancer (COU-AA-302): Final overall survival analysis of a randomised, double-blind, placebo-controlled phase 3 study. *The Lancet Oncology*.

[17] Penson D. F., Armstrong A. J., Concepcion R. (2016). Enzalutamide versus bicalutamide in castration-resistant prostate cancer: The STRIVE trial. *Journal of Clinical Oncology*.

[18] Beer T. M., Armstrong A. J., Rathkopf D. E. (2014). Enzalutamide in metastatic prostate cancer before chemotherapy. *The New England Journal of Medicine*.

[19] Maughan B. L., Luber B., Nadal R., Antonarakis E. S. (2017). Comparing Sequencing of Abiraterone and Enzalutamide in Men With Metastatic Castration-Resistant Prostate Cancer: A Retrospective Study. *The Prostate*.

[20] Eisenhauer E. A., Therasse P., Bogaerts J. (2009). New response evaluation criteria in solid tumours: revised RECIST guideline (version 1.1). *European Journal of Cancer*.

[21] Scher H. I., Halabi S., Tannock I. (2008). Design and end points of clinical trials for patients with progressive prostate cancer and castrate levels of testosterone: Recommendations of the Prostate Cancer Clinical Trials Working Group. *Journal of Clinical Oncology*.

[22] Cock P. J. A., Antao T., Chang J. T. (2009). Biopython: freely available python tools for computational molecular biology and bioinformatics. *Bioinformatics*.

[23] Viechtbauer W. (2010). Conducting meta-analyses in R with the metafor. * Journal of Statistical Software *.

[24] Cortés J., González J. A., Campbell M. J., Cobo E. (2014). A hazard ratio was estimated by a ratio of median survival times, but with considerable uncertainty. *Journal of Clinical Epidemiology*.

[25] Chopra A., Georgieva M., Lopes G., Yeo C. M., Haaland B. (2017). Abiraterone or Enzalutamide in Advanced Castration-Resistant Prostate Cancer: An Indirect Comparison. *The Prostate*.

[26] Partin A. W., Kattan M. W., Subong E. N. P. (1997). Combination of prostate-specific antigen, clinical stage, and Gleason score to predict pathological stage of localized prostate cancer: A multi-institutional update. *Journal of the American Medical Association*.

[27] Terada N., Maughan B. L., Akamatsu S. (2017). Exploring the optimal sequence of abiraterone and enzalutamide in patients with chemotherapy-naïve castration-resistant prostate cancer: The Kyoto-Baltimore collaboration. *International Journal of Urology*.

[28] Khalaf D., Annala M., Beja K. (2017). A randomized phase II cross-over study of abiraterone + prednisone (ABI) vs enzalutamide (ENZ) for patients (pts) with metastatic, castration-resistant prostate cancer (mCRPC). *Journal of Clinical Oncology*.

[29] Palapattu G. S. (2016). Commentary on “AR-V7 and resistance to enzalutamide and abiraterone in prostate cancer.” Antonarakis ES, Lu C, Wang H, Luber B, Nakazawa M, Roeser JC, Chen Y, Mohammad TA, Chen Y, Fedor HL, Lotan TL, Zheng Q, De Marzo AM, Isaacs JT, Isaacs WB, Nadal R, Paller CJ, Denmeade SR, Carducci MA, Eisenberger MA, Luo J, Division of Urologic Oncology, Department of Urology, University of Michigan, MI. N Engl J Med 2014; 371(11):1028-38.. *Urologic Oncology: Seminars and Original Investigations*.

[30] Nakazawa M., Lu C., Chen Y. (2015). Serial blood-based analysis of AR-V7 in men with advanced prostate cancer. *Annals of Oncology*.

[31] Antonarakis E. S., Lu C., Luber B. (2015). Androgen receptor splice variant 7 and efficacy of taxane chemotherapy in patients with metastatic castration-resistant prostate cancer. *JAMA Oncology*.

