# Supplementary material for: Efficacy of Abiraterone and Enzalutamide in Pre- and Postdocetaxel Castration-Resistant Prostate Cancer: A Trial-Level Meta-Analysis
Source: Prostate Cancer. 2017 Nov 21;2017:8560827. doi: 10.1155/2017/8560827 (PMC5735648; doi:10.1155/2017/8560827)
Supplement: Supplementary file 1 — Supplemental 1, Figure: Funnel plots showing the effect against precision for all treatment sequences for OS/PFS. Supplemental 2/3, Figure: Pearson correlations between median OS/PFS and other variables shown on the lower left with corresponding pairwise plots on the upper right. Distributions of each variable are displayed along the diagonal. Plotting PSA decline, Gleason score, and baseline PSA against median OS/PFS indicate trends that are consistent with expected clinical outcomes. Supplemental 4/5, Appendix: Results of regression models for median OS/PFS as outcomes. Hash marks indicate the specific model. [file 8560827.f1.pdf]

S1: Funnel Plots

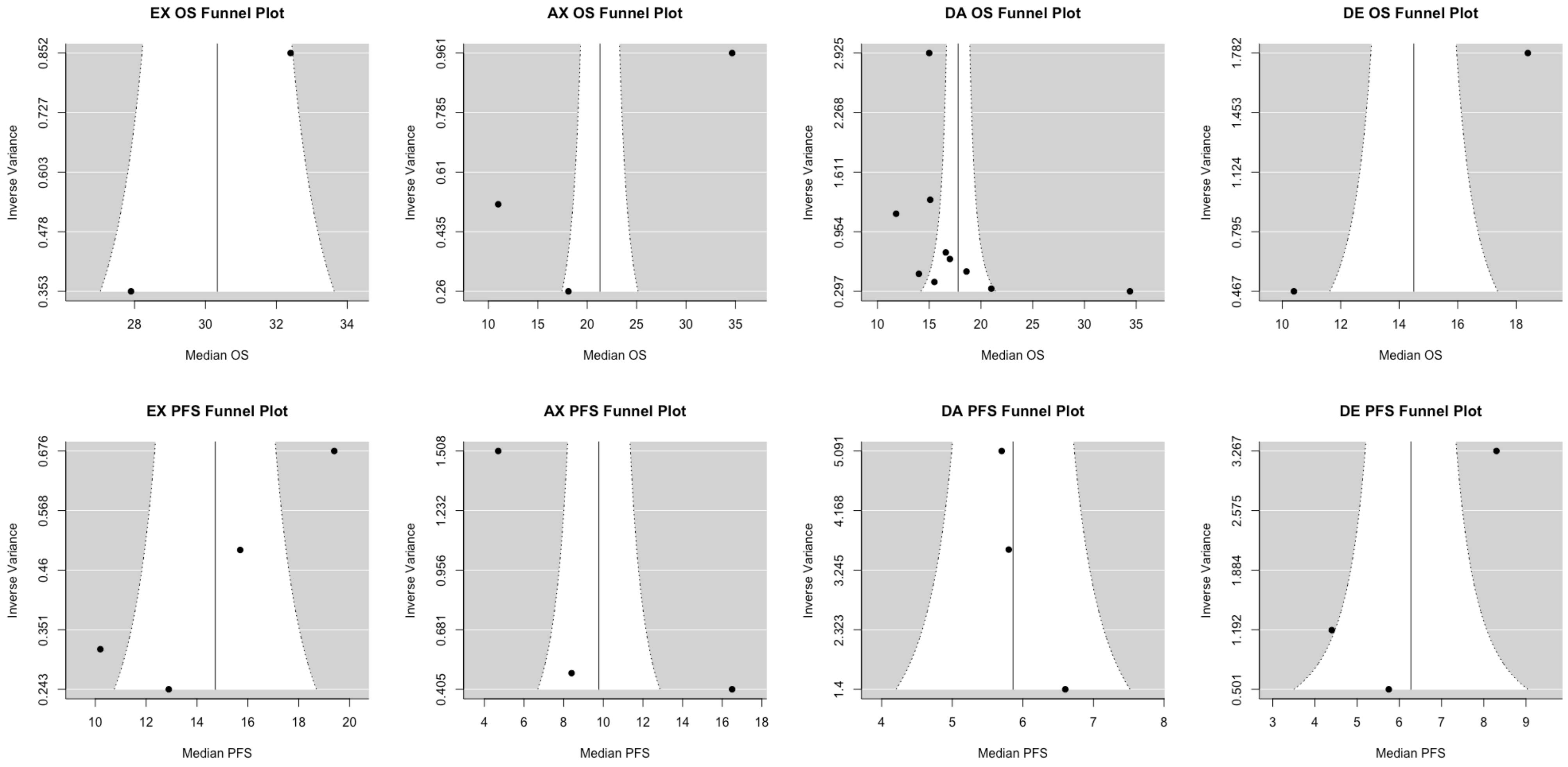

S2: Studies Reporting OS Estimates

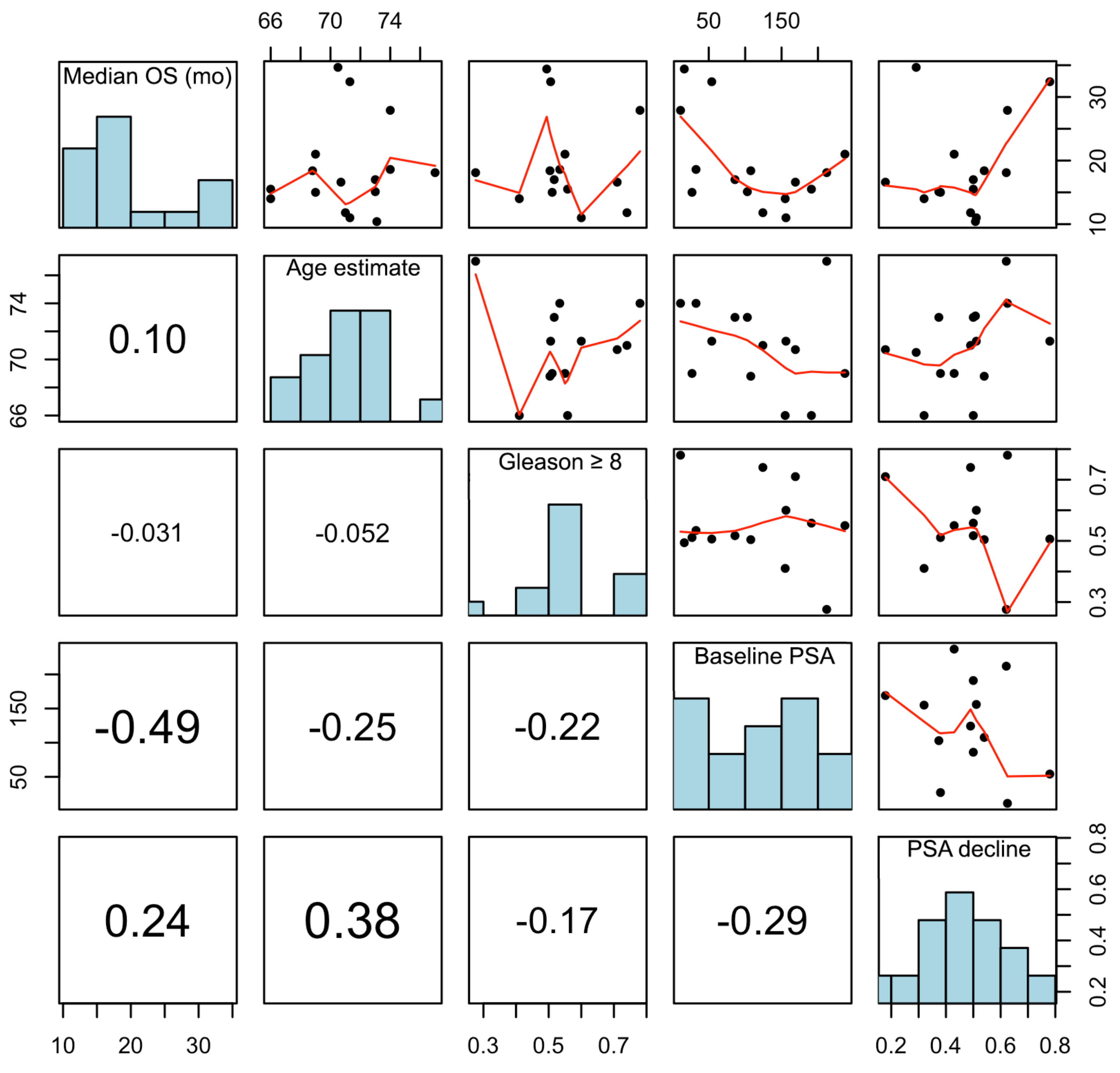

S3: Studies Reporting PFS Estimates

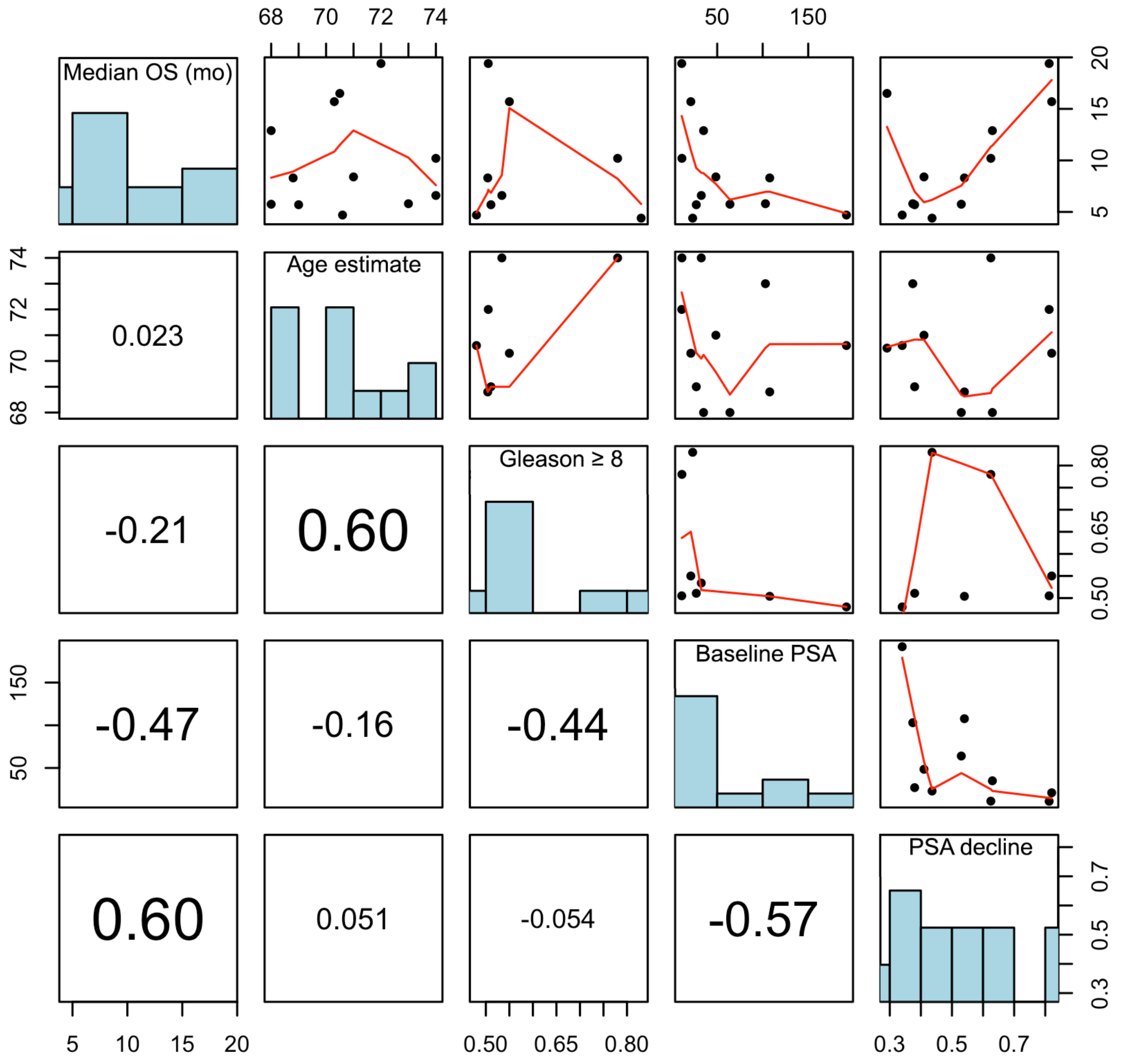

S4: Regression Analysis for Median OS

```
################################### mOS ~ gleason_8
            estimate      se     zval    pval     ci.lb    ci.ub
intrcpt      22.9784  1.6158  14.2214  <.0001   19.8116  26.1453  ***
gleason_8    -9.2563  2.8756  -3.2189  0.0013  -14.8924  -3.6202   **
################################### mOS ~ PSA_baseline
               estimate      se     zval    pval    ci.lb    ci.ub
intrcpt         20.1097  0.5241  38.3728  <.0001  19.0826  21.1369  ***
PSA_baseline    -0.0273  0.0048  -5.7313  <.0001  -0.0366  -0.0180  ***
################################### mOS ~ DAvDE
                      estimate      se     zval    pval    ci.lb    ci.ub
intrcpt                15.9322  0.3372  47.2455  <.0001  15.2713  16.5931  ***
factor(treatment)DE     0.8074  0.7473   1.0804  0.2799  -0.6573   2.2721
################################### mOS ~ AXvEX
                      estimate      se     zval    pval    ci.lb    ci.ub
intrcpt                25.1549  0.7589  33.1451  <.0001  23.6674  26.6423  ***
factor(treatment)EX     5.9267  1.1856   4.9989  <.0001   3.6030   8.2504  ***
################################### mOS ~ DAvDE + gleason_8
                      estimate      se     zval    pval     ci.lb    ci.ub
intrcpt                23.3303  2.0999  11.1102  <.0001   19.2146  27.4461  ***
factor(treatment)DE     1.5450  0.8625   1.7913  0.0732   -0.1455   3.2355    .
gleason_8             -12.8479  3.6631  -3.5074  0.0005  -20.0274  -5.6685  ***
################################### mOS ~ AXvEX + gleason_8
                      estimate      se     zval    pval     ci.lb    ci.ub
intrcpt                22.7763  2.8003   8.1335  <.0001   17.2878  28.2648  ***
factor(treatment)EX    19.5164  1.5371  12.6965  <.0001   16.5037  22.5292  ***
gleason_8             -19.1225  5.2077  -3.6720  0.0002  -29.3294  -8.9156  ***
```

S5: Regression Analysis for Median PFS

```
################################# mPFS ~ gleason_8
            estimate      se     zval    pval     ci.lb    ci.ub
intrcpt      10.0028  1.5122   6.6150  <.0001    7.0391  12.9666  ***
gleason_8    -4.9759  2.7427  -1.8142  0.0696  -10.3514   0.3997    .
################################# PFS ~ PSA_baseline
               estimate      se     zval    pval    ci.lb    ci.ub
intrcpt          8.1578  0.3874  21.0597  <.0001   7.3986   8.9170  ***
PSA_baseline    -0.0155  0.0045  -3.4794  0.0005  -0.0243  -0.0068  ***
################################# mPFS ~ DAvDE
                      estimate      se     zval    pval   ci.lb   ci.ub
intrcpt                 5.8608  0.3154  18.5838  <.0001  5.2426  6.4789  ***
factor(treatment)DE     1.2467  0.5489   2.2713  0.0231  0.1709  2.3225    *
################################# mPFS ~ AXvEX
                      estimate      se     zval    pval   ci.lb    ci.ub
intrcpt                 7.4394  0.6465  11.5069  <.0001  6.1722   8.7065  ***
factor(treatment)EX     8.3058  0.9978   8.3245  <.0001  6.3503  10.2614  ***
################################# mPFS ~ DAvDE + gleason_8
                      estimate      se     zval    pval     ci.lb    ci.ub
intrcpt                11.8987  1.7348   6.8587  <.0001    8.4985  15.2989  ***
factor(treatment)DE     2.2405  0.6627   3.3810  0.0007    0.9417   3.5393  ***
gleason_8             -11.6412  3.2762  -3.5533  0.0004  -18.0624  -5.2201  ***
################################# mPFS ~ AXvEX + gleason_8
                      estimate      se     zval    pval     ci.lb     ci.ub
intrcpt                19.8814  3.7832   5.2552  <.0001   12.4665   27.2964  ***
factor(treatment)EX    14.6263  1.3819  10.5843  <.0001   11.9179   17.3348  ***
gleason_8             -31.6280  7.6969  -4.1092  <.0001  -46.7136  -16.5423  ***
```
